# Inferring B cell specificity for vaccines using a Bayesian mixture model

**DOI:** 10.1186/s12864-020-6571-7

**Published:** 2020-02-22

**Authors:** Anna Fowler, Jacob D. Galson, Johannes Trück, Dominic F. Kelly, Gerton Lunter

**Affiliations:** 1 Department of Biostatistics, University of Liverpool, Liverpool, UK; 2 University Children’s Hospital Zurich and the Children’s Research Center, University of Zurich, Zurich, Switzerland; 3 Oxford Vaccine Group, Department of Paediatrics, University of Oxford and the NIHR Oxford Biomedical Research Center, Oxford, UK; 4 MRC Weatherall Institute of Molecular Medicine, University of Oxford, John Radcliffe Hospital, Oxford, UK

**Keywords:** B cell receptor, Vaccination, Immune repertoire, High-throughput sequencing

## Abstract

**Background:**

Vaccines have greatly reduced the burden of infectious disease, ranking in their impact on global health second only after clean water. Most vaccines confer protection by the production of antibodies with binding affinity for the antigen, which is the main effector function of B cells. This results in short term changes in the B cell receptor (BCR) repertoire when an immune response is launched, and long term changes when immunity is conferred. Analysis of antibodies in serum is usually used to evaluate vaccine response, however this is limited and therefore the investigation of the BCR repertoire provides far more detail for the analysis of vaccine response.

**Results:**

Here, we introduce a novel Bayesian model to describe the observed distribution of BCR sequences and the pattern of sharing across time and between individuals, with the goal to identify vaccine-specific BCRs. We use data from two studies to assess the model and estimate that we can identify vaccine-specific BCRs with 69% sensitivity.

**Conclusion:**

Our results demonstrate that statistical modelling can capture patterns associated with vaccine response and identify vaccine specific B cells in a range of different data sets. Additionally, the B cells we identify as vaccine specific show greater levels of sequence similarity than expected, suggesting that there are additional signals of vaccine response, not currently considered, which could improve the identification of vaccine specific B cells.

## Background

The array of potential foreign antigens that the human immune system must provide protection against is vast, and an individual’s B cell receptor (BCR) repertoire is correspondingly huge; it is estimated that a human adult has over 10^13^ theoretically possible BCRs [1], of which as many as 10^11^ may be realized [2]. This diversity is primarily generated through recombination, junctional diversity, and somatic mutation of the V, D and J segments of the immunoglobulin heavy chain genes (IgH) [2], combined with selection to avoid self-reactivity and to increase antigen specificity. The BCR repertoire of a healthy individual is constantly evolving, through the generation of novel naive B cells, and by the maturation and activation of B cells stimulated by ongoing challenges of pathogens and other antigens. As a result, an individual’s BCR repertoire is unique and dynamic, and is influenced by age, health and infection history as well as genetic background [3].

Upon stimulation, B cells undergo a process of proliferation and hyper-mutation, resulting in the selection of clones with improved antigen binding and ability to mount an effective immune response. The process of hypermutation targets specific regions, and subsequent selection provides a further focusing of sequence changes. The short genomic region in which most of these changes occur, and which is thought to play a key role in determining antigen binding specificity, is termed the Complementarity Determining Region 3 (CDR3) [4, 5]. Next generation sequencing (NGS) makes it possible to capture the CDR3 across a large sample of cells, providing a sparse but high-resolution snapshot of the BCR repertoire, and forming a starting point to study immune response and B-cell-mediated disease [6].

Vaccination provides a controlled and easily administered stimulus that can be used to study this complex system [7]. An increase in clonality has been observed in the post-vaccination BCR repertoire, which has been related to the proliferation of B cells and the production of active plasma cells [8–14]. An increase in the sequences shared between individuals, referred to as the public repertoire or stereotyped BCRs, has also been observed, and there is mounting evidence that this public repertoire is at least partly due to convergent evolution in different individuals responding to the same stimulus [10, 14–18].

These observations suggest that by identifying similarities between the BCR repertoires of a group of individuals that have received a vaccine stimulus, it may be possible to identify B cells specific to the vaccine. However, while the most conspicuous of these signals could be shown to be likely due to a convergent response to the same antigen in multiple individuals [19], it is much harder to link more subtle signals to vaccine response using ad-hoc classification methods. To address this, we here develop a statistical model for the abundance of BCRs over time in multiple individuals, which integrates the signals of increased expression, clonality, and sharing across individuals. We use this model to classify BCRs into three classes depending on the inferred states of their B cell hosts, namely non-responders (background, *bg*), those responding to a stimulus other than the vaccine (non-specific, *ns*), and those responding to the vaccine (vaccine-specific, *vs*).

Here we show that the sequences classified as vaccine-specific by our model have distinct time profiles and patterns of sharing between individuals, and are enriched for sequences derived from B cells that were experimentally enriched for vaccine specificity. Moreover, we show that sequences identified as vaccine-specific cluster in large groups of high sequence similarity, a pattern that is not seen in otherwise similar sets of sequences.

## Results

### Hepatitis B data set

**Fig. 1:**
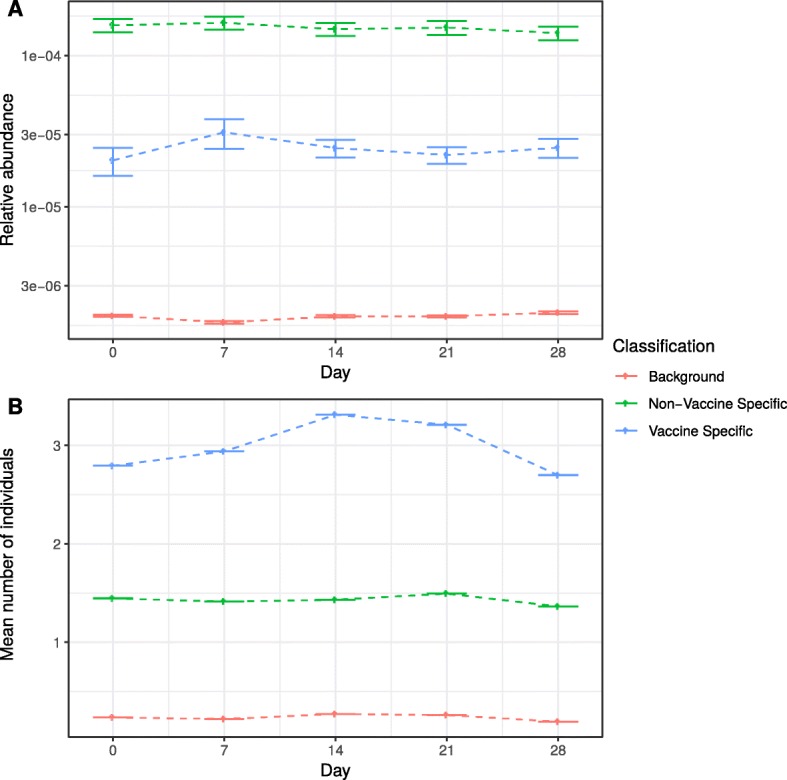
Temporal features of the hepatitis B data set by classification. Mean clonal relative abundance at each time point in each classification (**a**), and the mean number of individuals sharing a BCR clone over time in each classification (**b**) for the hepatitis B data set

A total of 1,034,622 clones were identified in this data set, with a mean total abundance of 6.7 (s.d. 419) with the largest clone containing 230,493 sequences across all samples and time points. We fitted the model to the hepatitis B data set, with key parameter estimates given in Table 1. Model fit was assessed using a simulation study, in which data was randomly generated from the generative model itself using the inferred parameters (Table 1). The simulated sequence abundance distributions follow the observations reasonably well (see Fig. 1; Additional file 1), despite these distributions being highly complex and heavy-tailed due to the complexity of the underlying biology. Thus, although the model simplifies many biological processes, the simulation suggests that it does effectively capture the underlying distributions from which the data arise.

**Table 1:** Fitted parameters to the hepatitis B data set

	*Γ*_*class*_			*p*_*class*_		*ω*_*class*_	
Class	*bg*	*ns*	*vs*	*bg*; *ns*	*vs*	*bg*; *vs*_*t*=0_	*ns*; *vs*_*t*>0_
	.992	.005	.003	.216	.970	.006	.277

*Γ*, the probability of a BCR belonging to each class; *p*, the probability of a BCR from each class being observed in an individual; *ω*, the probability of an observed BCR in each class being seen at high abundance

The value of *Γ*_*class*_ show that most BCRs are assigned to the background population, with only a small fraction responding to any stimuli. (This is also seen from the numbers shown in Table 2.) BCR clones classified as vaccine specific are highly likely to be shared between multiple individuals, reflected in a high estimate of *p*_*vs*_, and the high estimate of *ω*_*vs*_ mean they are also more likely to be seen at high frequencies than those classified as background.

**Table 2:** Number of sequences allocated to each category across all samples and the mean total sequence abundance across all samples, in the whole data set and in the subset also labelled as HBsAG+

**Classification**	**All BCR clones**		**HBsAG+ BCR clones**	
	Number	Abundance (sd)	Number	Abundance (sd)
Background	1,026,523	2.62 (31)	60,215	3.45 (44)
Non-specific	5123	89.3 (748)	1500	147.1 (1,084)
Vaccine-specific	2976	10.8 (174)	2055	10.7 (190)

For each of the three classes, the relative abundance of those clones within individuals and the number of individuals sharing them over time are illustrated in Fig. 1. The vaccine specific clones are seen at lower frequencies at day 0 compared to subsequent time points, but still at higher frequencies than sequences classified as background. The number of individuals sharing the vaccine specific clones increases over time up to a peak at day 14 after which sharing declines again, whereas in the other classes there is no significant trend in sharing across time points, as expected.

The total number of BCR clones allocated to each class and the mean total abundance of clones from all samples within each class are shown in Table 2. BCRs are overwhelmingly classified as background, while of the remainder, similar numbers are classified as non-specific responders and vaccine-specific responders. Clones classified as background all have very low abundance, often consisting of a single sequence observed in a single individual at a single time point. BCRs classified as non-specific form the largest clones, and are often seen at high abundance across all time points.

We next compared the hepatitis B data set with the HBsAG+ data to validate our results and provide an estimate of sensitivity. BCR clones from the hepatitis B data set were considered present in the HBsAG+ data set if there is a BCR in the HBsAG+ data which would be assigned to it. The number of clones from the hepatitis B data set that are present in the HBsAG+ data set, along with their abundances, are also given in Table 2. 60,215 (5.9%) of the clones classified as background were also present in the HBsAg+ data set, however a much larger fraction (69%) of those classified as vaccine-specific were also seen in the HBsAG+ dataset.

Although providing the nearest available approximation to a truth-set, the HBsAG+ data set contains a large number of erroneously captured cells, with the specificity of staining estimated to be around 50% [20]. These erroneously captured cells are likely to be those present in high abundance in the whole repertoire (and therefore in the hepatitis B data set) due to random chance. The difference in enrichment between the background and vaccine specific categories will therefore be partly driven by the different average abundance of background clones (2.62) compared to vaccine-specific clones (10.8). However, the fraction of non-specific responders observed in the HBsAG+ set (29%) is intermediate between that of background and vaccine-specific clones, despite non-specific responders having a substantially larger average abundance than clones from either of these classes (89.3), indicating that the method is capturing a subset that is truly enriched with vaccine-specific clones.

The average abundance of all clones classified as vaccine specific which are also found in HBsAG+ is similar to the average abundance of all vaccine specific clones (10.7 in comparison to 10.8). In contrast, in the background and non-specific categories, the average abundance is far higher for those clones which are also present in the HBsAG+ data set (an increase from 2.62 to 3.45 in background clones, and 89.3 to 147.1 in vaccine specific clones). This further suggests that the clones identified as vaccine specific which are also found in the HBsAG+ data set are truly binding the antigen rather than being selected at random with a size bias.

**Fig. 2:**
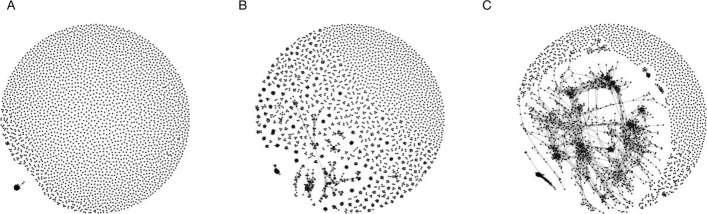
Petri-plots of hepatitis B data set by classification. Similarity between BCR sequences classified as background (**a**), non-specific response (**b**), and vaccine-specific (**c**). Each point corresponds to a clone; clones are connected if the Levenshtein distance between their representative CDR3 sequences is less than *n*/5 where *n* is the sequence length. All vaccine-specific BCR sequences are shown and a length-matched, random sample of the same number of sequences from the background and non-specific sequences are shown

We next looked at sequence similarity *between* clones within each class. Using the Levenshtein distance, we found that clones classified as vaccine specific had CDR3 sequences were significantly more similar to each other than those of clones classified as background (*p*<0.001 based on 1,000 simulations; Fig. 2; Additional file 1). This is further illustrated in petri-dish plots (Fig. 2); here clonal centres were connected by edges if their Levenshtein distance was less than 20% of the sequence length in order to highlight the greater degree of sequence similarity in vaccine specific sequences. Vaccine specific clones show cliques, and filament structures suggestive of directional selection, while non-responders and particularly background clones show much less between-clone similarity.

For comparison, we also applied the thresholding method to this data set and the criteria for clones to be considered vaccine specific varied. Clones classified as vaccine specific using this method were then compared to the HBsAG+ sequences and the percentage agreement reported. A range of different criteria were tried, and those which demonstrate how the choice of threshold affect results, as well as ones found to be optimal, are shown in Table 3. The strictest threshold, requiring clonal abundance to be in the top.01 quantile at any time point post-vaccination and in the bottom.99 quantile pre-vaccination as well as requiring that sequences are shared between at least 3 individuals, has the highest percentage of sequences which are also in the HBsAG+ data set. Increasing the sharing threshold from 1 to 3 individuals dramatically increases the percentage of clones which are also in the HBsAG+ data set, indicating that the requirement of seeing sequences in multiple individuals is important. The agreement with the HBsAG+ data set (on which estimates of sensitivity are based) is much lower using this approach than using the model we’ve developed; the highest estimate of sensitivity we obtained using thresholding is 53.7% whereas with out model we estimate it to be 69%.

**Table 3:** Clones classified as vaccine specific using different threshold abundance and sharing criteria

Abundance threshold	Shared	Number of clones	Number of sequences	HBsAG+ agreement
.9	1	54,334	1,743,271	12.1%
.9	3	5609	396,354	47.1%
.99	1	5221	1,475,448	23.3%
.99	3	1097	505,536	53.7%

### Influenza data set

**Fig. 3:**
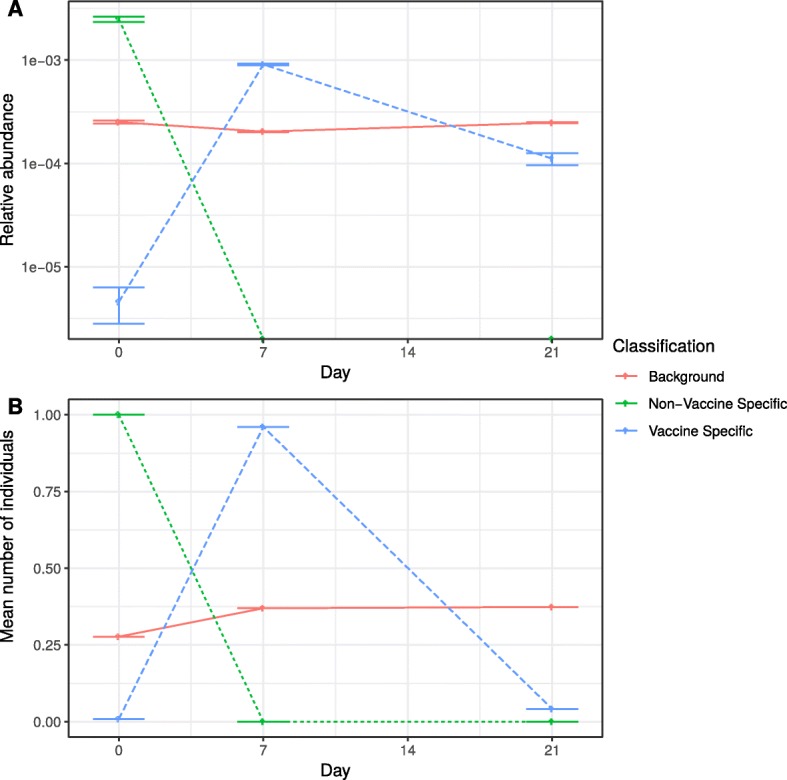
Temporal features of the influenza data set by classification. Mean clonal relative abundance at each time point in each classification (**a**), and the mean number of individuals sharing a clone over time in each classification (**b**) for the influenza data set

A total of 28,606 clones were identified in this data set, with an mean abundance of 1.5 (s.d. 1.3) with the largest clone containing 86 sequences across all samples and time points. Fitting the model to the Influenza data set, we again obtain a good QQ plot (see Fig. 3; Additional file 1) indicating an acceptable model fit, despite considerable differences in the two data sets. Key parameter estimates and an overview of the classification results are given in Tables 4 and 5, and again show that most clones are classified as belonging to the background population, with only a small fraction classified as responding to any stimuli. However, in this data set, clones classified as vaccine specific are no more likely to be seen in multiple individuals than those classified as background. Another difference is that the model assigns vanishing weight to the possibility that background clones are observed at high abundance.

**Table 4:** Fitted parameters to the influenza data set

	*Γ*_*class*_			*p*_*class*_		*ω*_*class*_	
class	*bg*	*ns*	*vs*	*bg*; *ns*	*vs*	*bg*; *vs*_*t*=0_	*ns*; *vs*_*t*>0_
	.947	.001	.051	.144	.144	0	.486

**Table 5:** Number of clones allocated to each category across all samples, the mean total clonal abundance across all samples, and number of sequences also found in the plasmablast data set from each classification

**Classification**	**All clones**		**Plasmablast**
	Number	Abundance (sd)	Number
Background	27,120	1.45 (1.06)	11
Non-specific	23	5.52 (0.85)	0
Vaccine-specific	1463	2.51 (1.54)	3

The clonal abundance and number of individuals sharing clones over time are illustrated in Fig. 3, for each classification. The vaccine specific clones show a distinct sequence abundance profile, with a sharp increase post-vaccination which reduces over time, whereas the background clones show little change over time. The average number of individuals sharing a clone is below one for all categories at all time points, indicating that most clones are only seen in single individuals and not at multiple time points.

The number of clones allocated to each class and the clonal abundance within each class are shown in Table 5. The majority of clones are classified as background with a small number being classified as vaccine specific, and only 23 classified as being part of a non-specific response. The clones classified as vaccine-specific are also typically more abundant.

We then compared the sequences in the influenza data set to those obtained from plasmablasts collected post vaccination, an approximate truth-set of sequnces which are likely to be vaccine-specific. Again, a sequence from the influenza data set was considered to be present in the plasmablast data set if there exists a clone in the plasmablast data set to which it would be assigned (Table 2). Of the 436 sequences in the plasmablast data set, 14 are found to be present in the influenza data set, of which 3 would be classified as vaccine specific. These results are considerably less striking as for the hepatitis B data set, although vaccine-specific clones are still borderline significantly enriched within the monoclonal antibody sequences compared to background clones (*p*=0.03, two-tailed Chi-squared test).

**Fig. 4:**
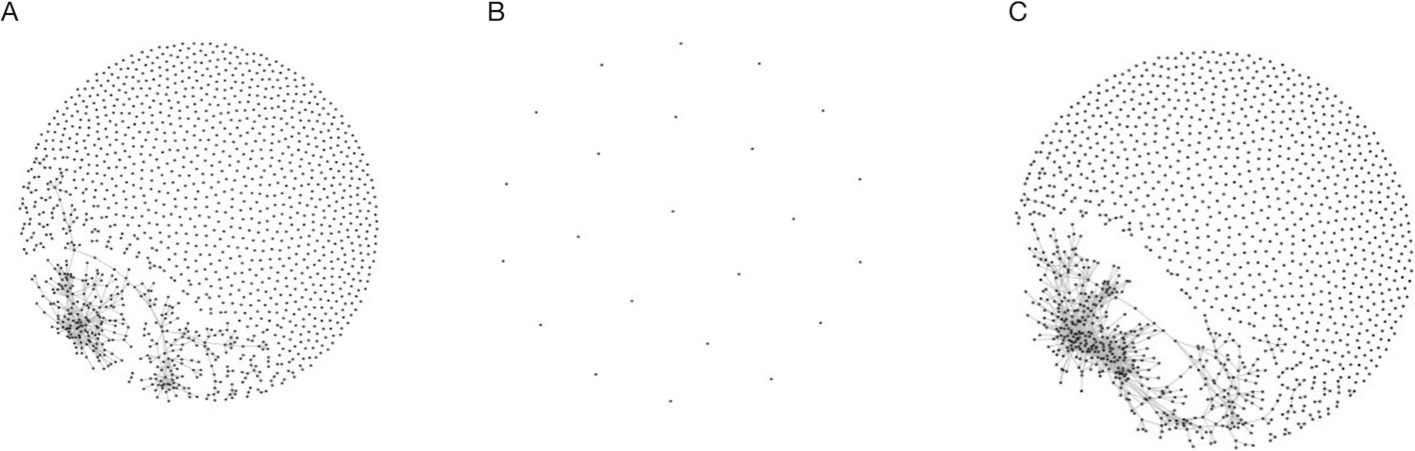
Petri-plots of hepatitis B data set by classification. Similarity between BCR sequences classified as background (**a**), non-specific response (**b**), and vaccine-specific (**c**). Each point corresponds to a clone; clones are connected if the Levenshtein distance between their representative CDR3 sequences is less than *n*/3 where *n* is the sequence length. All vaccine-specific and non-specific BCR sequences are shown and a random sample from the background sequence, which is length and size matched with the vaccine-specific sequences, is shown

The clones classified as vaccine specific in the influenza data set were also found to be more similar than expected by random chance (*p*<0.001 based on 1,000 simulations; see Fig. 4; Additional file 1). This is illustrated in Fig. 4 in which clones (represented by points) are joined if the Levenshtein distance between their CDR3 sequences is less than *n*/3, where *n* is the sequence length. Note that this threshold was chosen to highlight the greater sequence similarity present in vaccine specific sequences and is more stringent than that used for the hepatitis B data set because the viral data consist of amino acid sequences.

For comparison, we also applied the thresholding method to this data set and the criteria for clones to be considered vaccine specific varied. Clones classified as vaccine specific using this method were then compared to the plasmablast sequences and the percentage agreement reported, although it is worth noting that there is only a small number of plasmablast sequences so this doesn’t represent an estimate of accuracy but does provide a means of comparison between different threshold values and with the modelling approach. A range of criteria were tried, and results which demonstrate the effect of changing the criteria, along with the optimal criteria tried, are shown in Table 6. The lowest threshold, requiring clonal abundance to be in the top.1 quantile at any time point post-vaccination and in the bottom.9 quantile pre-vaccination as well as only requiring that clones are seen in one individual, has the highest percentage of sequences which are also in the plasmablast data set. However, even the threshold parameters with the highest percentage agreement with the plasmablast data set only share a single sequence, whereas our modelling approach shares three sequences. The thresholding parameters which are optimal according to the agreement with the plasmablast data set are very different to the optimal thresholding parameters for the HepB data set and mirror the parameter estimates learnt using our model.

**Table 6:** Clones classified as vaccine specific using different threshold abundance and sharing criteria

Abundance threshold	Shared	Number of clones	Number of sequences	Plasmablast agreement
.9	1	1,294	5,666	0.1%
.9	3	15	184	0%
.99	1	134	1,171	0%
.99	3	5	95	0%

## Discussion

Vaccine specific BCRs are identified with an estimated 69% sensitivity, based on clones classified as vaccine specific in the hepatitis B data set and their concordance with sequences experimentally identified as vaccine specific in the HBsAG+ data set. The HBsAG+ data set is more likely to contain those clones present in high abundance in the whole repertoire, due to random chance and a relatively low specificity. This is reflected in the clones classified as background and as non-specific, in which the average abundance seen in these categories and in the HBsAG+ data set is higher than the average abundance of all clones in these categories. However, this over representation of highly abundant sequences is not seen in the clones classified as vaccine specific, suggesting they are indeed binding the vaccine and supporting our estimate of sensitivity.

The influenza data set was compared to the set of sequences from plasmablasts collected post vaccination. However, only 14 of these plasmablast sequences were identified in the influenza set making any estimate of sensitivity from this data set unreliable. Of these plasmablast sequences, 21% were classified as vaccine specific; this is a similar amount to those identified by [10] as in clonally expanded lineages and therefore likely to be responding to the vaccine.

This model incorporates both the signal of clonal abundance as well as sharing between individuals. The thresholding approach indicates the importance of each of these signals by allowing us to vary them independently. It demonstrates that for the HepB data set, sensitivity (estimated through agreement with the HBsAG+ data set) is increased by at least 30% by including a sharing criteria of clones being seen in at least 3 individuals. Conversely, the thresholding method also shows that for the influenza data set, including a shared criteria reduces the agreement with the plasmablast data set of clones which are likely to be responding to the vaccine. The parameters inferred using the modelling approach also reflect the importance of sharing in the different data sets, and allow us to automatically learn this from the data.

Although the clones we identify as vaccine specific are often highly abundant, their average abundance is modest, with the non-specific response category containing the most abundant clones. Similarly whilst some clones identified as vaccine specific were shared between multiple individuals, many were only seen in a single participant. It is only by combining these two signals through the use of a flexible model that we are able to identify the more subtle signatures of vaccine response.

We see evidence for convergent evolution in the hepatitis B data set, with clones identified as vaccine specific being much more likely to be seen in multiple individuals. Despite a convergent response to the influenza vaccine being observed by others [10, 17], this pattern is not seen in the influenza data set, in which the probability of a vaccine specific sequence being observed in an individual is similar to that for the background sequences. There are several potential explanations for this. Firstly, in the influenza data set, the signal of sharing among individuals may have been overwhelmed by the abundance signal; many more potentially vaccine specific cells are identified here than in previous studies. Secondly, the influenza data set captures a smaller number of sequences from DNA, whereas the hepatitis B data set captures a larger number of sequences from RNA, so there may be less sharing present in the influenza data set in part due to random chance and in part due to the lack of over-representation of highly activated (often plasma cells) B cells. Thirdly, the hepatitis B vaccine was administered as a booster whereas the influenza was a primary inoculation, therefore some optimisation of the vaccine antigen binding is likely to have already occurred after the initial hepatitis B vaccine, increasing the chance that independent individuals converge upon the same optimal antigen binding. Lastly, the complexity of binding epitopes of either of the vaccines is unknown, and the lack of convergent evolution could be explained by a much higher epitope complexity of the influenza vaccine compared to that of the hepatitis B vaccine. This would result in a more diffuse immune response on the BCR repertoire level, making it harder to identify.

In both the hepatitis B and the influenza data sets, it is likely that the sequences show more underlying structure than is accounted for using our clonal identification approach which only considers highly similar sequences of the same length. The CDR3 sequences from clones identified as vaccine specific show greater similarity than expected by random chance when utilising the Levenshtein distance, which allows for sequences of different lengths. A possible explanation for this is that there could be a motif shared between sequences of different lengths which could be driving binding specificity. It is possible that by allowing for more complex similarity relationships, larger groups which are more obviously responding to the vaccine may emerge, however current methods are too computationally intensive to allow for complex comparisons of all sequences from all samples.

Here we focus on the signals of clonal abundance and sharing between individuals to identify sequences from vaccine specific clones. The flexibility of the model allows for data sets to be analysed which differed in vaccination strategy, sampling time points, sequencing platforms and nucleic acids targeted. However there are many clones which are likely incorrectly classified, for instance since random PCR bias can result in large numbers of sequences, if these occur in samples taken at the peak of the vaccine response, they would likely be incorrectly labelled as vaccine specific. Alternatively, vaccination may trigger a non-specific B cell response, B cells involved in this response would have an abundance profile which follows that expected of sequences responding to the vaccine and would therefore likely be misclassified. The inclusion of additional signals, such as hyper-mutation, would improve our model and our estimates of sensitivity.

## Conclusion

The B cell response to vaccination is complex and is typically captured in individuals who are also exposed to multiple other stimuli. Therefore distinguishing B cells responding to the vaccine from the many other B cells responding to other stimuli or not responding at all is challenging. We introduce a model that aims to describe patterns of clonal abundance over time, convergent evolution in different individuals, and the sampling process of B cells, most of which occur at low abundance, from BCR sequences generated pre- and post-vaccination. These patterns are different between B cells that respond to the vaccine stimulus, B cells that respond to a stimulus other than the vaccine, and the bulk of non-responding B cells. By using a mixture model to describe the pattern of clonal abundance for each of these cases separately, we are able to classify BCRs as either background, non-specific or vaccine specific. In comparison to existing, thresholding methods, our method provides far higher sensitivity in comparison to a ‘truth set’ of sequences enriched for those which are vaccine specific. Additionally, our method is able to automatically determine the optimal parameters, rather than having to specify criteria for thresholding which is difficult when little is known about how much these criteria differ across data sets.

## Methods

### BCR repertoire vaccine study data sets

We use two publicly available data sets, one from a study involving a hepatitis-B vaccine [20] and one from a study on an influenza vaccine [10]. We describe these two data sets below. Both data sets capture the somatically rearranged VDJ region in B cells, in particular the highly variable CDR3 region on which we will focus.

#### Hepatitis B

In the study by Galson and colleagues [20], 5 subjects were given a booster vaccine against hepatitis B (HepB) following an earlier primary course of HepB vaccination. Samples were taken on days 0, 7, 14, 21 and 28 relative to the day of vaccination. Total B cells were sorted and sequenced in all samples. We refer to this data set as the *hepatitis B data set*.

In addition, cells were sorted for HepB surface antigen specificity at the same time points post-vaccination. The mRNA that was reverse transcribed to cDNA in these cells was then amplified using Vh and isotype specific primers and these IgH transcripts were then sequenced. These cells are enriched with those we are seeking to identify using our modelling approach, and provides the nearest available approximation to a truth-set of sequences which are vaccine-specific. We refer to these data as the *HBsAG+ data set*. Both data sets are publicly available on the Short Read Archive (accession PRJNA308641).

Sequences were generated on the Illumina platform using an RNA sequencing protocol, and the nucleotide sequences analysed. Targeting RNA means that highly abundant sequences may derive either from multiple B cells from a clonal subpopulation, or from one or a small number of B cells with high IgH gene expression, such as plasma cells that are actively secreting antibodies. Although we cannot distinguish between these two possibilities, both classes of cells are likely signifiers of immune response, and are therefore of interest.

#### Influenza

We also analyze data from subjects that were vaccinated against influenza in a study by Jackson and colleagues [10]. Samples were taken on days 0, 7 and 21 relative to vaccination. We analyzed a subset of 7 subjects that were deemed to be “seroconverters” who have an increased level of antibodies in response to the vaccine, based on vaccine-specific ELISA assays. This will be referred to as the *influenza data set*.

In addition, the authors also collected plasmablasts on day 7 in 5 of the subjects. These are also likely to be enriched for B cells responding to the vaccine and therefore act as an approximate truth-set providing an additional source of evaluation for our method. The sequences derived from these cells are referred to as the *plasmablast data set*. All data is publicly available on dbGaP (accession phs000760.v1.p1).

The Roche 454 platform was used to perform DNA sequencing of the somatically recombined IgH locus, using primers for the relatively conserved FR2 IgH V gene segment, and a conserved IgH J gene segment [10], and we analyse the amino acid sequences. Targeting DNA ensures that sequences with high abundance are representative of clonally expanded B cells, rather than of cells exhibiting high mRNA expression. However, active plasma cells with high secretion rate would still be counted individually.

### Clonal identification

We combined sequences into *clones* primarily to group together sequences arising from the same clonal expansion, and this also serves to correct for read errors and group together some highly similar sequences that likely target the same epitope. This removes some noise associated with read error and strengthens signals by treating multiple sequences all of which target the same epitope as a single clone, whilst also reducing the computational burden. Each clone consists of a single identifying CDR3 sequence, the *clonal centre*, and its set of neighbouring CDR3 sequences; for two sequences to be considered neighbours, they must be of the same length and be highly similar, which we define as greater than 85% similarity for nucleotide sequences as in the hepatitis B data set, or 90% similarity for amino acid sequences as in the influenza data set. The clonal identification was performed in a greedy manner, by iteratively identifying a clonal centre as the sequence with the greatest number of neighbours from among all unassigned sequences, and assigning it and its unassigned neighbours to a new clone. This is a computationally efficient approach to clonal identification which allows us to process very large data sets. However, the model presented here is not dependent on the clonal identification method used, and any alternative method could also be used as input.

Within each data set, we identified clones using all samples and time points together, but kept track of sample- and time-specific counts to enable the analysis of time dynamics and between-individual sharing. This results in some clones which are present in multiple individuals and therefore considered ‘public’ clones. We now consider each clone to be representative of the BCR sequence *i* at its centre, and make no distinction between clones and the individual sequences which form the clonal centres. In addition we shall use *i* to refer to the B cell(s) that the clone represents. We define the *clonal abundance*, denoted by *x*_*ist*_, as the number of sequences assigned to clone *i* for a participant *s* at time point *t*, and the total clonal abundance as the total number of sequences assigned to the clone across all samples, $\sum _{st}x_{ist}$.

### Model

We introduce a hierarchical Bayesian model to describe the clonal abundance (or alternatively, CDR3 sequences) across individuals inoculated with the same vaccine, and across multiple time points. The data are abundances, *x*_*ist*_, as introduced above. The goal of modeling these data is to identify CDR3 sequences of vaccine-specific BCR clones from among a large number of non-vaccine-specific BCRs, while accounting for sparse sampling and for the highly stochastic nature of the biological process that generates them.

One identifying feature of vaccine-specific BCR clones that we want to model is their abundance profile. We expect to observe no vaccine-specific BCRs pre-vaccination (or very few, in the case of a primer-boost design such as for the HepB data set), while post-vaccination we expect to observe high abundances due to clonal expansion of stimulated B cells, the presence of plasma cells with high transcription activity, or both. A second feature that helps to characterise vaccine-specific BCRs is their tendency to be shared across individuals, due to convergent evolution.

To describe the model we introduce some notation. As above let *i* denote a BCR clone, and denote by *Ω* the space of all clones. We partition this set as *Ω*=*Ω*_*bg*_∪*Ω*_*vs*_∪*Ω*_*ns*_, where the disjunct subsets represent background BCR clones not responding to any stimulus; vaccine-specific BCR clones responding to the vaccine stimulus; and BCR clones responding to a non-specific stimulus other than the vaccine respectively. These subsets (and their sizes) are unknown, and the classification of a particular clone *i* is given by a discrete random variable *γ*_*i*_∈{*bg*,*vs*,*ns*}, so that $i \in \Omega _{\gamma _{i}}$.

Next, the presence of a particular B cell clone *i* in a participant *s* is encoded by a second discrete random variable *z*_*is*_, which takes on the value 0 when *i* is absent from the BCR repertoire of individual *s* at any time point, and 1 when *i* is present in the individual (though not necessarily present in any sample taken from this individual). The variable *z* aims to account for the sparsity resulting from the diversity of BCR repertoires from different individuals. The distribution of *z*_*is*_ is dependent on *γ*_*i*_, to allow modeling the increased probability that vaccine-specific BCRs are shared between individuals.

**Fig. 5:**
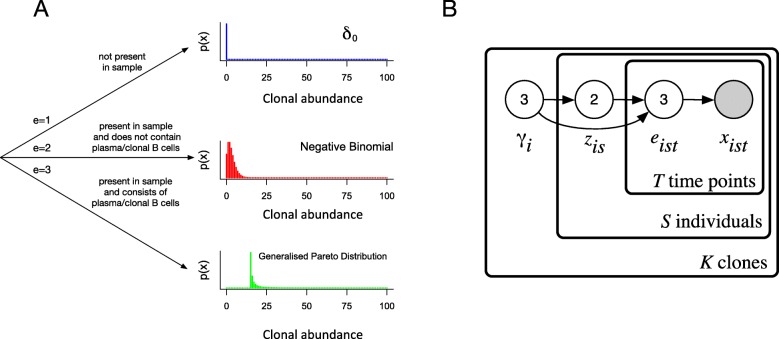
Model diagrams. **a** Tree diagram in which each leaf represents a generative distribution for clonal abundances. The probability of following each path is dependent on the classification of the BCR clone and the presence of the sequence in the individual. **B** Partial graphical representation of the model using plate notation. For clarity, hyperparameters are not show; Fig. 5; Additional file 1 contains a complete diagram

The actual abundances *x*_*ist*_ of clone *i* in individual *s* at a time point *t* are assumed to be independent conditional on *γ*_*i*_ and *z*_*is*_, and are modeled by a mixture of three distributions representing three outcomes, modeled by a third discrete random variable *e*_*ist*_ whose distribution depends on *γ*_*i*_, *z*_*is*_ and *t*. First, the relevant B cell or cells may be absent from individual *s* (if *z*_*is*_=0) or may have escaped sampling. In this case *x*_*ist*_ is distributed as a point mass at 0. Second, if B cells have been sampled, they may be neither clonal nor plasma B cells, and would therefore contribute a small number of sequences to the data set. In this case *x*_*ist*_ is modeled as a negative Binomial distribution. The remaining case is that the sampled B cell or cells are either plasma cells, or cells sampled from a large clonal population (or both), in which case they are expected to contribute a large number of sequences. In this case *x*_*ist*_ is modeled as a discretised generalized Pareto distribution [21]. This distribution of abundances is illustrated in Fig. 5a. The mixture distribution of clonal abundance *x*_*ist*_ is given by *p*(*x*_*ist*_|*e*_*ist*_,***θ***), where ***θ*** is the vector of parameters of the negative Binomial and generalized Pareto distributions.

The resulting joint probability for a data set ***x***, latent variables ***e***,***z*** and parameters ***γ***,***θ*** under this model is given by

1 $$ {\begin{aligned} p(\boldsymbol{\theta},\boldsymbol{\gamma},\boldsymbol{z},\boldsymbol{e},\boldsymbol{x}) & = p(\boldsymbol{\theta}) \prod_{i} p(\gamma_{i}) \prod_{s}p(z_{is}|\gamma_{i}) \prod_{t} p(e_{ist}|\gamma_{i},z_{is},t)p(x_{ist}|e_{ist},\boldsymbol{\theta}) \end{aligned}} $$

The relationship between the variables in the model is shown in Fig. 5b. Non-informative priors *p*(***θ***) and *p*(*γ*) are placed on the parameters; this allows these parameters to be learnt from the data, and therefore allows the model to be applied to a range of data sets, for instance RNA sequencing and DNA sequencing. Full details of the model and priors are provided in the “Background” section; Additional file 1.

We restrict *i* to range over only those BCRs which are observed at least once in the data set, rather than the 10^13^ that are theoretically possible. Therefore, for *K* BCR clones, we have that 1≤*i*≤*K*. This simplifies model fitting, but will result in parameter estimates which are specific to each individual data set, and therefore affected by features such as the number of individuals. This should be kept in mind when interpreting the results.

### Inference

The model is fitted to each data set using an Expectation-Maximisation (E-M) algorithm which iteratively maximises the model allocation parameters conditional on the parameters which determine the distribution of each classification, and vice versa; see Additional file 1 for details. Initial parameters were chosen to reflect our prior beliefs that clones responding to the vaccine would be more likely to be present in low abundance pre-vaccination but high abundance post vaccination, and that they are more likely to be seen in multiple individuals, and results were robust to initial values which preserve these beliefs. This approach ensures that the parameters associated with each class are consistent with its biological interpretation and avoids the problem of label switching. Since these data sets are particularly large, and the number of model parameters relatively small, there is little uncertainty in our parameter estimates. Therefore, this approach is a computationally efficient alternative to Markov Chain Monte Carlo (MCMC) approaches, which is able to optimise the posterior.

Restrictions on parameter values allow us to encode additional structure and to link parameters hierarchically. Firstly we assume that there is no time-dependence for the abundances of B cells classified as background or as non-specific responders. We further assume that for the vaccine-specific cells, the pre-vaccination abundances (at *t*=0) follow the same distribution as B cells classified as background, while post-vaccination these cells follow the same abundance distribution as B cells classified as non-specific responders. Third, we assume that the probability of a clone being observed in a subject is the same for B cells classified as background and those classified as a non-specific response. In effect this assumes that non-specific responders are or have been responding to private stimuli, rather than for instance earlier common infections.

The uncertainty in the inferred model parameters is negligible in comparison to the biological noise because of the large amount of data. Rather than reporting this spurious precision, we report the parameter estimates without error bars, but we note that errors due to model misspecification are likely to be substantial. We report the inferred probability of a BCR clone belonging to each category, *Γ*_*class*_ for $\phantom {\dot {i}\!}class \in \{\mathsf {bg},\mathsf {vs},\mathsf {ns}\}$. We also report, for each class, the probability that a clone is observed given that a corresponding B cell of that class is present in an individual, *p*_*class*_. Finally, we report for each class the inferred probability that a clone is being observed with high abundance, *ω*_*class*_.

### Sequence similarity

To compare the within-set similarity of sequences between subsets of sequences of any length, we use the Levenshtein (or “edit”) distance as implemented in [22]. Specifically, given a subset of sequences, we compute a measure of within-set similarity the mean of the Levenshtein distances between all pairs of sequences in the subset. To assess significance we use bootstrapping: we calculate the mean Levenshtein distance between a randomly selected subset of the same size, and compare the resulting null distribution of means to calculate the empirical p-value.

### Thresholding method

Existing methods for identifying vaccine specific BCR clones rely on identifying sequences which are either highly abundant, shared between multiple individuals, or both. Empirical methods are typically used to determine thresholding criteria for abundance and sharing [8, 18, 20], sequences which are above these thresholds are then considred to be likely vaccine specific. Alternatively, statistical significance of sequences in cases relative to controls can be used to determine threshold levels [16], or training and test sets used for validation [19].

We define an abundance threshold above which clones are considered to be highly abundant as a quantile of all abundances in an individual sample [20]. This allows the actual abundance value to change according to sample variability such as sequencing depth. Clones may then be considered vaccine-specific if they are below this threshold pre-vaccination and above this threshold for at least one time point post-vaccination. We also define a sharing threshold as the minimum number of individuals in which a clone must be present in order to be considered vaccine specific [19]. Sequences from individual clones are considered vaccine-specific if both the abundance and sharing criteria are met, and we evaluate a range of different thresholds by comparing them to our truth sets.

## Supplementary information

**Additional file 1** Supplementary materials. Contains additional details of the model and plots of results.

## Data Availability

The data sets analysed here are publicly available. The hep B data set is available on the Short Read Archive, accession PRJNA308641, and the influenza data set is available on dbGaP accession phs000760.v.1.p1. The code is available at https://github.com/AnnaFowler/BCell-VaccineInference.git.

## Abbreviations

BCR: B cell receptor
CDR3: Complementarity determining region 3
E-M: Expectation-maximisation
HepB: Hepatitis B
IgH: Immunoglobulin heavy chain
MCMC: Markov chain monte carlo
NGS: Next generation sequencing
