# Supplementary material for: Inferring B cell specificity for vaccines using a Bayesian mixture model
Source: BMC Genomics. 2020 Feb 22;21:176. doi: 10.1186/s12864-020-6571-7 (PMC7036227; doi:10.1186/s12864-020-6571-7)
Supplement: Supplementary file 1 — Additional file 1 Supplementary materials. Contains additional details of the model and plots of results. [file 12864_2020_6571_MOESM1_ESM.pdf]

Inferring B cell specificity for vaccines using a Bayesian mixture model

Supplementary materials

Anna Fowler, Jacob D. Galson, Johannes Trück, Dominic F. Kelly and Gerton Lunter

1 Model

For a dataset $\boldsymbol{x}$ consisting of clonal abundances in subjects, s and at time points, t, the joint probability of the model is built up conditionally, and given by:

$$p(\boldsymbol{\theta}, \boldsymbol{\gamma}, \boldsymbol{z}, \boldsymbol{e}, \boldsymbol{x}) = p(\boldsymbol{\theta}) \prod_i p(\gamma_i) \prod_s p(z_{is}|\gamma_i) \prod_t p(e_{ist}|\gamma_i, z_{is}, t) p(x_{ist}|e_{ist}, \boldsymbol{\theta}) \quad (1)$$

where $\boldsymbol{\gamma}$ is the latent allocation vector denoting the allocation of BCR clones to classes (background, vaccine specific or non-vaccine specific); $\boldsymbol{z}$ is a binary variable indicating the presence or absence of a clone within an individual; and $\boldsymbol{e}$ is the latent allocation vector denoting the underlying distribution from which the clonal abundances are generated.

The parameter $\boldsymbol{e}$ is not of primary interest, so we marginalise over it, and obtain a posterior which is equivalent to a mixture model:

$$p(\boldsymbol{\gamma}, \boldsymbol{z}, \boldsymbol{\theta}|\boldsymbol{x}) \propto p(\boldsymbol{\theta}) \prod_i p(\gamma_i) \prod_s p(z_{is}|\gamma_i) \prod_t \sum_{\eta=1}^{3} p(e_{ist} = \eta|\gamma_i, \zeta, t) p(x_{ist}|\eta, \boldsymbol{\theta}) \quad (2)$$

The vector $\boldsymbol{\theta} = \boldsymbol{\theta}_{1,1}, \ldots, \boldsymbol{\theta}_{ST}$ contains the sample specific parameters associated with the underlying clonal abundance distributions, where

$$p(x_{ist}|\eta, \boldsymbol{\theta}) = \begin{cases} 0 & \text{if } \eta = 1 \\ NB(x_{ist}|\boldsymbol{\theta}_{st}) & \text{if } \eta = 2 \\ dGPD(x_{ist}|\boldsymbol{\theta}_{st}) & \text{if } \eta = 3 \end{cases} \quad (3)$$

where NB is the density of the negative-binomial distribution and $dGPD$ is the density of the discretised Generalised Pareto Distribution [1]. These parameters are subject and time point dependent allowing for differences between the samples, in particular sequencing depths. The dGPD has a threshold parameter, and only assigns probability to values above this threshold. This ensures that it is only capturing the tail of the distribution (those clones which are seen in high abundance) and provides an intuitive interpretation that only clones seen at abundances above this threshold could be considered clonal.

We adopt a flexible approach allowing the model to be applied to a range of data sets, and therefore we use non-informative priors and seek to learn parameters as much as possible. We choose Dirichlet priors for the distribution

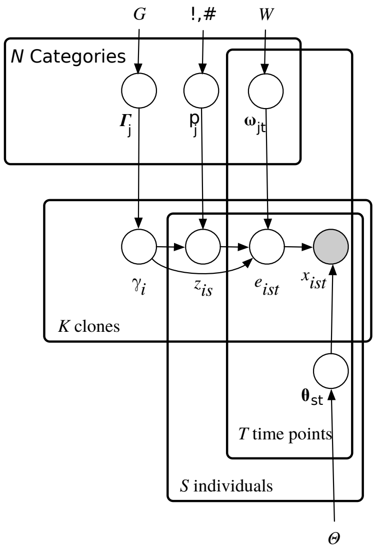

Figure 1: Full graphical representation of model using plate notation.

of γ_i and e_{ist}, and a Beta prior for z_{is}; more precisely,

$$\begin{aligned}
p(\gamma_i = class) &= \Gamma_{class} \quad \text{for } 1 \leq i \leq K;\ class \in \{bg, vs, ns\} \\
\Gamma_{class} &\sim Dir(G) \\
p(z_{is} = 1|\gamma_i) &\sim Bernouilli(p_{\gamma_i}) \quad \text{for all } s \\
p_{\gamma_i} &\sim Beta(\alpha, \beta) \\
p(e_{ist}|\gamma_i, z_{is}, t) &= \omega_{\gamma_i,t} \quad \text{for all } s \\
\omega_{\gamma_i,t} &\sim Dir(W) \\
\boldsymbol{\theta} &\sim Unif(\Theta),
\end{aligned}$$

where K is the number of clones and Dir is the symmetric Dirichlet distribution. We set $G = W = 1$ to give the flat Dirichlet distribution, $\alpha = \beta = 1$ to give a uniform distribution, and Θ defines the space of all possible parameter values. The full model is illustrated in plate notation in Figure 1.

1.1 Inference

The parameters are fitted to the data sets using an E-M algorithm. Initial parameter values are based on prior belief that vaccine specific clones will be rare, seen at high frequency and shared between multiple samples, and the results are robust to different initial parameter values which maintain these properties. This choice of initial parameters was seen to prevent problems of label switching and to identify clones with properties typically associated with vaccine response, whilst allowing the data to inform the final parameter values.

Restrictions on parameter values allow us to encode additional structure and to link parameters hierarchically. First, we assume no structure in the time profile for the B cell abundances which are not responding to the vaccine, so that $\omega_{\mathsf{bg},t} = \omega_{\mathsf{bg}}$ and $\omega_{\mathsf{ns},t} = \omega_{ns}$ for all t. The time profile that we assume for the vaccine-specific cells assumes that pre-vaccination the abundances of vaccine-specific cells have the same distribution as the background cells ($\omega_{\mathsf{vs},0} = \omega_{\mathsf{bg}}$), and that post-vaccination they have the same abundance distribution as B cells responding to a stimulus other than the vaccine ($\omega_{\mathsf{vs},t} = \omega_{\mathsf{ns}}$, for $t > 0$). We also assume that the probability of a clone being observed in a subject is the same for B cells classified as background and those classified as a non-specific response, that is, $p_{\mathsf{bg}} = p_{\mathsf{ns}}$ Finally, $z_{is} = 0$ indicates an absence of B cells in subject, so in this case we restrict the B cell abundance to being generated by the point mass at zero by defining $p(e_{ist} = 1|\gamma_i, z_{is} = 0, t) = 1$.

In order to prevent convergence to degenerative local maxima we restrict $\Gamma_{class} \geq .001$, so that is there is always some small probability of a clone belonging to any class.

2 Hepatitis B Q-Q plot

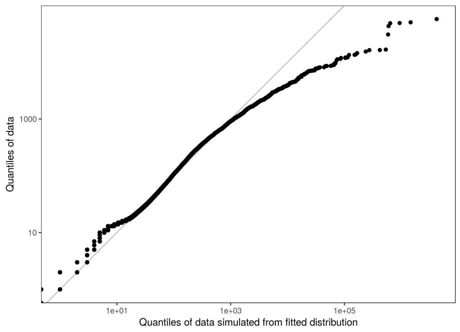

Figure 2: Log-scale Q-Q plots of clonal abundances, conditional on clones being present in an individual, and data simulated from the fitted distribution for each sample. This complex data set with a heavy tail is well represented by the fitted distribution.

3 Hepatitis B simulated p-value

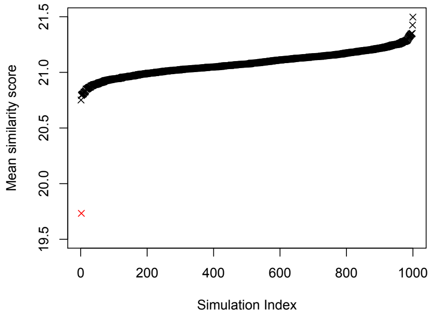

Figure 3: The mean Levenshtein distance between all pairs of sequences, in a random subset (black), and the vaccine specific subset (red) in the Hep B data set.

4 Influenza Q-Q plot

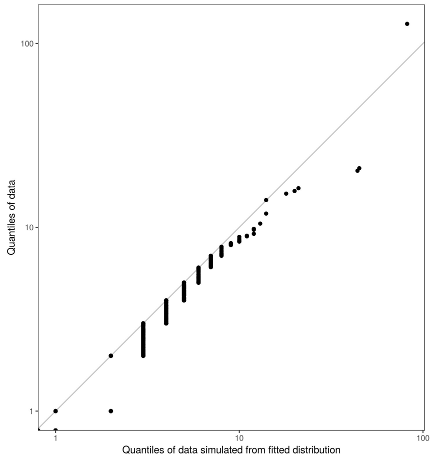

Figure 4: Log-scale Q-Q plots of clonal abundance, conditional on clones being present in an individual, and data simulated from the fitted distribution for each sample. This complex data set with a very heavy tail is fitted reasonably well by this distribution.

5 Influenza simulated p-value

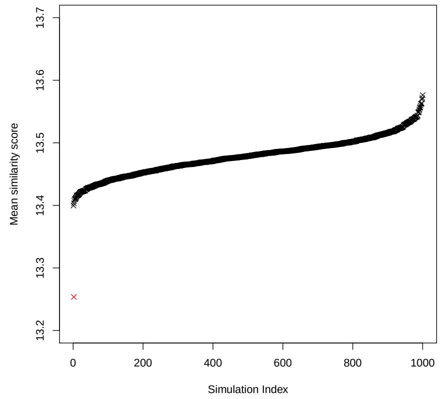

Figure 5: The mean Levenshtein distance between all pairs of sequences, in a random, length-matched, subset (black), and the vaccine specific subset (red) in the Influenza data set.

References

[1] Amrutha Buddana and Tomasz J Kozubowski. Discrete pareto distributions. *Stochastics and Quality Control*, 29(2):143–156, 2014.
